# Interaction of (+)-Strebloside and Its Derivatives with Na^+^/K^+^-ATPase and Other Targets

**DOI:** 10.3390/molecules26185675

**Published:** 2021-09-18

**Authors:** Yulin Ren, Sijin Wu, Sijie Chen, Joanna E. Burdette, Xiaolin Cheng, A. Douglas Kinghorn

**Affiliations:** 1 Division of Medicinal Chemistry and Pharmacognosy, College of Pharmacy, The Ohio State University, Columbus, OH 43210, USA; ren.41@osu.edu (Y.R.); wusj@dicp.ac.cn (S.W.); chen.3428@buckeyemail.osu.edu (S.C.); 2 Department of Pharmaceutical Sciences, College of Pharmacy, University of Illinois at Chicago, Chicago, IL 60612, USA; joannab@uic.edu

**Keywords:** (+)-strebloside, cytotoxicity, Na^+^/K^+^-ATPase, docking profiles, molecular targets

## Abstract

Docking profiles for (+)-strebloside, a cytotoxic cardiac glycoside identified from *Streblus asper*, and some of its derivatives and Na^+^/K^+^-ATPase have been investigated. In addition, binding between (+)-strebloside and its aglycone, strophanthidin, and several of their other molecular targets, including FIH-1, HDAC, KEAP1 and MDM2 (negative regulators of Nrf2 and p53, respectively), NF-κB, and PI3K and Akt1, have been inspected and compared with those for digoxin and its aglycone, digoxigenin. The results showed that (+)-strebloside, digoxin, and their aglycones bind to KEAP1 and MDM2, while (+)-strebloside, strophanthidin, and digoxigenin dock to the active pocket of PI3K, and (+)-strebloside and digoxin interact with FIH-1. Thus, these cardiac glycosides could directly target HIF-1, Nrf2, and p53 protein–protein interactions, Na^+^/K^+^-ATPase, and PI3K to mediate their antitumor activity. Overall, (+)-strebloside seems more promising than digoxin for the development of potential anticancer agents.

## 1. Introduction

*Streblus asper* Lour. (Moraceae) is a medium-sized tree used in several systems of traditional medicine and shows multiple bioactivities, including antimicrobial [1], antihepatitis [2], and antitumor activities [3]. Currently, the potential antitumor activity of *S. asper* is attracting wide interest, and cardiac glycosides have been identified as the major active components [4,5,6], of which the content was found to vary among different parts of this plant [7].

(+)-Strebloside (**1**) was originally isolated as a major cardiac glycoside from the root bark of *S. asper* [8], following which its NMR spectroscopic data were assigned [4]. The absolute configuration of **1** has been determined by analysis of its ECD and NMR spectra and by comparison of these spectroscopic data with those of digoxin, for which the structure was confirmed using single-crystal X-ray diffraction data [9]. In an earlier study, (+)-strebloside (**1**) was found to show potent cytotoxicity against HeLa human cervical cancer cells (ED_50_ 0.06 µM) [4], and it was demonstrated recently to exhibit activity toward other human cancer cells [5,9,10]. Interestingly, (+)-strebloside was found to show more potent cytotoxicity (IC_50_ 0.17 and 0.11 µM against the human HT-29 colon and H1299 non-small cell lung cancer cell lines, respectively) than digoxin (IC_50_ 0.38 µM against HT-29 cells and 0.46 µM toward H1299 cells) [9]. In an in vivo hollow fiber assay, (+)-strebloside displayed activity when immunodeficient NCr *nu*/*nu* mice implanted with hollow fibers containing MDA-MB-231 or OVCAR3 cells were treated (i.p.) daily with this compound at doses of 5.0 mg/kg for four days, but no obvious side effects were observed in the mice treated with this compound, even at the high dose of 30 mg/kg used [9]. Mechanistic investigations showed that (+)-strebloside mediates its potential antitumor activity by inhibition of Na^+^/K^+^-ATPase (NKA) and nuclear factor kappa B (NF-κB) activation, induction of cancer cell apoptosis, and inhibiting potently mutant p53 expression through the induction of the extracellular signal-regulated kinase (ERK) pathway [11], although it does not affect glucose transport in HT-29 human colon cancer cells [10]. However, to the best of our knowledge, detailed information about the interaction of this compound and its potential molecular targets has not been reported yet.

Cardiac glycosides are important natural products, of which several compounds have long been used to treat congestive heart failure, and some of these were found more recently to show anticancer activity. However, the narrow therapeutic index, limited selectivity, and lack of promise shown thus far in cancer clinical trials have become major challenges for the development of these compounds as anticancer agents [12], and these restrictions were found to correlate with their NKA inhibition [13]. Interestingly, (+)-strebloside was found to show limited toxicity in mice, and it mediates antitumor activity by targeting apoptosis induction, NKA, NF-κB, and p53 [9,11]. Thus, investigation of the interactions between (+)-strebloside and its molecular targets could be supportive of the development of new anticancer agents from this compound.

Docking profiles for digoxin and selected derivatives and NKA and other targets have been reported, and digoxin was postulated to interact directly with FIH-1, NF-κB, and NKA to mediate its antitumor activity [14]. Following these previous investigations, the binding between NKA and (+)-strebloside (**1**) and its derivatives (**2**–**11**), in which the structure of **1** has been changed at the C-3, C-10, and C-17 positions, has been investigated herein. In addition, the correlation between the docking scores and the cytotoxicity of **1**–**11** and the docking profiles for (+)-strebloside (**1**), digoxin, or their aglycones and factor inhibiting HIF-1 (FIH-1), histone deacetylase (HDAC), mouse double-minute 2 protein [MDM2, a negative regulator of tumor protein p53 (p53)], Kelch-like ECH-associated protein 1 [KEAP1, a negative regulator of nuclear factor erythroid 2-related factor 2 (Nrf2)], NF-κB, phosphoinositide 3-kinase (PI3K) and Akt1 (Protein kinase B1) is discussed. 

## 2. Results

### 2.1. Impact of the C-3 Saccharide Moiety and the C-10 Formyl Group on the Binding between (+)-Strebloside and NKA

**Figure 1:**
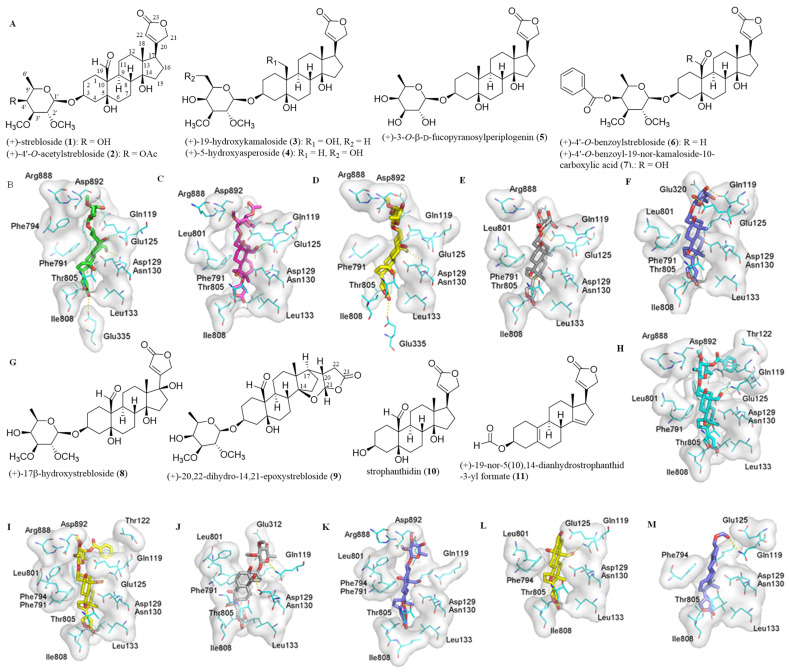
Structures of (+)-strebloside (**1**), (+)-4′-*O*-acetylstrebloside (**2**), (+)-19-hydroxykamaloside (**3**), (+)-5-hydroxyasperoside (**4**), (+)-3-*O*-β-D-fucopyranosylperiplogenin (**5**), (+)-4′-*O*-benzoylstrebloside (**6**), and (+)-4′-*O*-benzoyl-19-nor-kamaloside-10-carboxylic acid (**7**) (**A**) and (+)-17β-hydroxystrebloside (**8**), (+)-20,22-dihydro-14,21-epoxystrebloside (**9**), strophanthidin (**10**), and (+)-19-nor-5(10),14-dianhydrostrophanthidin-3-yl formate (**11**) (**G**) and docking profiles for **1** (green, **B**), **2** (pink, **C**), **3** (yellow, **D**), **4** (gray, **E**), **5** (blue, **F**), **6** (cyan, **H**), **7** (yellow, **I**), **8** (gray, **J**), **9** (blue, **K**), **10** (yellow, **L**), and **11** (blue, **M**) and NKA. The modeled structure of human NKA (hNKA) was used as the receptor, and the conformations of **1**–**11** generated by LigPrep were used in molecular docking against the receptor by AutoDock Vina [14].

Previously, all of the C-3 saccharide moiety, the C-10 formyl substituent, the C-5, C-14, and C-4′ hydroxy groups, the C-17 lactone unit, and the established conformation of (+)-strebloside (**1**) were found to be important in the mediation of its cytotoxicity against HT-29 human colon cancer cells and in its binding to NKA [9,10]. To test the role of the C-3 saccharide moiety and the C-10 formyl group in the interaction with NKA, **1** and several selected derivatives have been docked to the human NKA (hNKA) model that had been built in our previous study [14] using AutoDock Vina [15,16]. These derivatives included (+)-4′-*O*-acetylstrebloside (**2**), (+)-19-hydroxykamaloside (**3**), (+)-5-hydroxyasperoside (**4**), (+)-3-*O*-β-D-fucopyranosylperiplogenin (**5**), (+)-4′-*O*-benzoylstrebloside (**6**), and (+)-4′-*O*-benzoyl-19-nor-kamaloside-10-carboxylic acid (**7**) (Figure 1). The crystal structure of 4HYT (co-crystal structure of NKA and ouabain) has been used as a reference, with the docking scores and cancer cell cytotoxicity of these compounds being presented in Table 1.

When compared with the crystal structure of 4HYT, the binding pose of the steroid core of (+)-strebloside (**1**) was found to rotate about 45°. Such a rotation does not affect the orientation of the α-surface, but it makes the C-10 formyl group reside close to the αM2 helix to form a hydrogen bond with the Asn130 residue of NKA. Additionally, hydrogen bonds could be formed between the lactone unit and Glu335, between the C-5 hydroxy group and Gln119, and between the C-14 hydroxy group and Thr805. These hydrogen bonds strengthen the interaction between **1** and NKA, which is different from that of digoxin and this protein (Figure 1). However, the acetyl group at the C-4′ position of **2** forms a hydrogen bond with Glu320, which prohibits **2** from docking to the binding pocket as deeply as **1**, and the acetyl substitution of **2** results in the loss of several important hydrogen bonds observed for **1** (Figure 1). Thus, both the binding affinity and cytotoxicity of **2** are less potent than those of **1** (Table 1).

Compound **3** contains a hydroxymethyl rather than a formyl group at the C-10 position, and the hydrogen bond formed between the C-19 hydroxy group and the Asn130 residue of NKA could be weaker than that formed between the C-10 formyl group of **1** and this same residue. This weaker interaction leads to a slight rotation of the steroid core of **3** to break the hydrogen bond formed between the C-5 hydroxy group and the Gln119 side chain of NKA observed in **1** (Figure 1). However, the binding poses of **1**, **3**, and ouabain are closely similar (Figure 1), and thus the binding affinity and cytotoxicity of **3** do not vary greatly when compared with **1** (Table 1). Compound **4** has a methyl and a hydroxy group at its C-10 and C-5′ positions, respectively, and thus it is not able to form a hydrogen bond with Gln119 or Asn130, as observed for ouabain and **1**, respectively (Figure 1). Even though this compound may form a conserved hydrogen bond with the Thr805 residue, and its C-5′ hydroxy group is close to and forms a hydrogen bond with the sidechain of Glu125, these interactions prohibit **4** from docking to the cation binding site of NKA deeply. Thus, the interaction of **4** and NKA was weaker than that of **1** (Table 1). Similarly, two additional hydrogen bonds formed between the C-2′ and C-3′ hydroxy groups of **5** and the Glu124 and Glu125 residues of NKA, respectively, nearly compensate for the loss of hydrogen bonds formed between the C-10 formyl group of **1** and the Asn130 side chain of NKA, and thus the interaction of **5** and NKA was similar to that of **1** (Figure 1 and Table 1).

Both **6** and **7** have a benzoyl group substituted at the C-4′ position, and they could form several hydrogen bonds with the cation binding pocket of NKA, including those formed between their C-10 formyl and C-14 hydroxy groups and the respective Gln119 and Thr805 side chains. However, the benzoyl group of **6** has a tendency to insert into a small pocket formed by Ile118, Leu319, Ile323 and the main chain of Gln119. This leads the saccharide moiety of **6** to be close to the αM1 and αM4 helices, which results in the loss of several hydrogen bonds observed in **1** and NKA (Figure 1). The binding pose of **7** is similar to that of **6** (Figure 1), but its binding affinity is lower than **6** (Table 1). The C-10 carboxylic acid substituent of **7** is charged negatively under physiological conditions, and the charged oxygen is repulsive to the negatively charged residues Glu112 and Asp129. Even though hydrogen bonds could form between its C-10 carboxylic acid and the Glu125 and Gln119 side chains of NKA, the increased distances between the hydrogen bond acceptor and donor could diminish the hydrogen bonding interaction. Thus, the binding affinity of **7** is low, and it does not show any activity toward HT-29 human colon cancer cells (Table 1).

### 2.2. Impact of the Lactone Unit on the Binding between (+)-Strebloside and NKA

To test the importance of the C-17 unsaturated heterocyclic unit, the binding between (+)-strebloside derivatives containing different substituents at the C-17 position, such as (+)-17β-hydroxystrebloside (**8**) and (+)-20,22-dihydro-14,21-epoxystrebloside (**9**), and NKA was investigated. In contrast to **1**, compound **8** fits the cation binding site with at least three different poses, but they all break the hydrogen bond network observed between **1** and NKA (Figure 1). Thus, **8** binds to NKA less strongly than **1**, and it does not show cytotoxicity against HT-29 human colon cancer cells (Table 1). Compound **9** contains an epoxy group at the C-14 and C-21 positions, and its binding pose changes greatly, even though it still forms a hydrogen bond between the C-14 substituent and Thr805 (Figure 1). The varied orientation of the lactone unit of **9** impairs its interaction with NKA, and, as a result, greatly decreased binding affinity and cytotoxicity were observed for **9** (Table 1).

### 2.3. Binding between the Aglycone of (+)-Strebloside or its Analogue and NKA

Previous investigations have shown that the C-3 saccharide moiety plays an important role in binding between (+)-strebloside (**1**) and NKA [10], and the binding parameters between digoxin and its aglycone and NKA were found to be different [14]. Thus, the docking profiles for the aglycone of **1**, strophanthidin (**10**), and its analogue, (+)-19-nor-5(10),14-dianhydrostrophanthidin-3-yl formate (**11**), and NKA have been investigated herein (Figure 1). Since the saccharide moiety of (+)-strebloside (**1**) causes important conformational and interaction changes (Figure 1), the binding pose of **10** is different from that of **1**. However, the binding pose of **10** seems similar to that of ouabain (Figure 1), and several conserved hydrogen bonds could be formed between the C-3 hydroxy group, the C-10 formyl group, and the C-14 hydroxy group and the Glu125, Gln119, and Thr805 side chains of NKA, respectively. These hydrogen bonds may improve the binding affinity of **10** when compared with digoxigenin [14]. However, the absence of hydroxy groups at the C-5 and C-14 positions and the formyl group at the C-10 position in **11** results in the loss of the hydrogen bonds formed between these groups and the residues of NKA, as observed in **10**. Additionally, the less polar steroid moiety and the more rigid skeleton decrease the binding affinity of **11** substantially (Table 1), even though its binding pose is similar to that of ouabain (Figure 1).

The docking profiles for compounds **1**–**11** and NKA indicate the importance of the C-3 saccharide moiety and the C-10 formyl and the C-5, C-14, and C-4′ hydroxy groups, as well as the C-17 lactone unit in the interaction of **1** with NKA and in the mediation of its cancer cell cytotoxicity. These substituents affect binding between **1** and NKA by interfering with the formation of hydrogen bonds and docking to the Asn130 or other side pockets, and modification at these substituents could greatly change the binding between **1** and NKA. Interestingly, introducing a C-10 carboxylic acid substituent may result in a repulsive force on NKA, which substantially decreases the binding affinity of the compound.

### 2.4. Correlation between Cytotoxicity of ***1**–**11*** and the Docking Scores from their Binding to NKA

Using AutoDock Vina, the docking scores were calculated for the binding of compounds **1**–**11** and NKA (PDB entry 4RET, the complex of NKA E2P-digoxin with bound Mg^2+^), while the cytotoxicity of compounds **1**–**9** and **11** against the HT-29 human colon cancer cell line was reported by our group [9], with that toward A549 human lung cancer cells of **1** and **10** being published by other research teams [5,17]. As shown in Table 1, most of these compounds were found to bind to NKA, and the docking scores correlate well with their cancer cell cytotoxicity (Table 1). For example, all of the cytotoxic compounds (**1**–**6** and **10**) showed an overall lower docking score than the non-cytotoxic compounds (**7**–**9** and **11**). In addition, compound **1** showed a lower docking score and more potent cytotoxicity than **2**, and consistent trends were observed when comparing **1** with **5, 3** with **4,** and **5** with **6** (Table 1). These data are in agreement with those observed for digoxin and its derivatives, of which the docking scores correlated well with their cancer cell cytotoxicity [14]. Furthermore, correlation between the docking scores from binding to NKA and cancer cell cytotoxicity of compounds **1–11** presented in Table 1 has been tested by Spearman correlation, using the GraphPad Prism 6.0 program. This leads to a significant correlation between them, with r equal to 0.8506 and *p* = 0.0017 (two-tailed). Thus, these two types of data of **1–11** correlate with each other, and the docking score values can potentially be used in the prediction of cytotoxicity of cardiac glycosides when they target NKA directly.

### 2.5. Binding between (+)-Strebloside (***1***) or Its Aglycone, Strophanthidin (***10***), and NF-κB, KEAP1, and PI3K and Akt1

(+)-Strebloside has been proposed to potentially target not only NKA but also NF-κB and p53 to mediate its antitumor activity [11], and hence these proteins and several other related targets were selected in the present molecular docking investigation. In addition, the importance of the saccharide moiety in binding between cardiac glycosides and NKA was evidenced [18,19], and thus both (+)-strebloside (**1**) and its aglycone, strophanthidin (**10**), were selected as the representatives in our docking experiments.

NF-κB plays a key role in inflammation and human pathobiology, and inhibition of this protein could contribute to the treatment of autoimmune and lymphoproliferative disorders [20]. Previously, both **1** and **10** were found to inhibit the activation of the NF-κB pathway, indicating that these cardenolides may directly target NF-κB to mediate their bioactivity [11,21]. To test this hypothesis, compounds **1** and **10** were docked into the p50 (PDB entry 1NFK), the p52 (PDB entry 1A3Q), and the p65 (PDB entry 2RAM) subunits of NF-κB by AutoDock Vina, using our published procedure [14]. In the p50 and p52 subunits, **1** and **10** take similar binding poses, and the β-surface of the steroid core fits the cavity of the DNA binding site. However, the polar interaction between the hydroxy groups at the steroid core of **1** and **10** and the proteins is not as stable as the hydrophobic interaction between digoxin and these proteins, and thus neither **1** nor **10** could bind to these targets. In the p65 subunit, the binding pose of **1** and **10** is different from that in the p50 and p52 subunits. The docking pose of **1** is somewhat similar to that of digoxin, but its interaction varies slightly, owing to the ca. 90 °C rotation of its steroid core. While **10** tends to interact with the hinge region of the p65 subunit of NF-κB with several hydrogen bonds, these interactions are very weak. Thus, neither **1** nor **10** may bind to these proteins as digoxin does. Consistently, the docking scores calculated from the binding between **1** and **10** and NF-κB are higher than those from digoxin and these proteins (Table 2). Thus, **1** and **10** do not bind well to NF-κB, and the inhibitory effects on the protein observed for **1** and **10** could result from their inhibiting NF-κB signaling rather than from a direct interaction with these proteins.

Both NF-κB and Nrf2 act as master regulators of the response to oxidative stress and inflammation, and a crosstalk between these proteins has been evidenced [22,23]. For example, Nrf2 inhibits oxidative stress-mediated NF-κB, which decreases free cAMP response element binding protein (CBP), a transcriptional co-activator of Nrf2. In addition, NF-κB regulates the Nrf2-mediated expression of antioxidant response element (ARE) [24,25], and such a crosstalk affects the growth and survival of a malignant tumor [26]. KEAP1 negatively regulates Nrf2 when it binds to two Kelch subunits in the KEAP1 dimer, and inhibition of the KEAP1-Nrf2 protein–protein interaction could activate Nrf2 [27,28]. Thus, the NF-κB inhibitory compounds, **1** and **10**, were investigated for their interaction with KEAP1.

**Figure 2:**
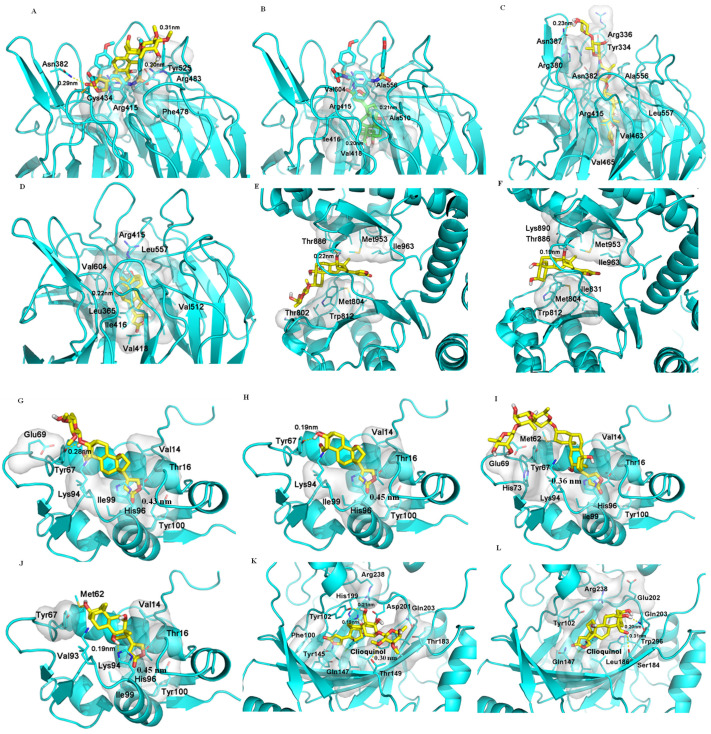
Docking profiles for (+)-strebloside (**1**, **A**), strophanthidin (**10**, **B**), digoxin (**C**), and digoxigenin (**D**) and KEAP1 (PDB entry 6V6Z), for **1** (**E**) and **10** (**F**) and PI3K (PDB: 6AUD), for **1** (**G**), **10** (**H**), digoxin (**I**), and digoxigenin (**J**) and MDM2 (PDB entry 4HBM), and for **1** (**K**) and **10** (**L**) and FIH-1 (PDB entry 3KCX). AutoDock Vina was used to generate the docking profiles, and the crystal structures of the target proteins were obtained from the Protein Data Bank (PDB) and employed as the receptors for the docking with **1**, **10**, digoxin, and digoxigenin [14], with the interaction distances being in the range 0.19–0.45 nm (H-bond distances: 0.19–0.23 nm).

When **1** and **10** were docked to KEAP1 (PDB entry 6V6Z), they both tend to stay at the entrance of the pocket to engage in polar interactions with the proteins, but they are not able to penetrate the cavity deeply. Hydrogen bonds are formed between **1** but not **10** and the Asn382, Arg483, and Ser555 residues of KEAP1. While **10** takes a pose similar to that of **1**, it also has a second pose by inserting into the cavity to interact with KEAP1, and this interesting binding pose is maintained entirely by a hydrophobic interaction (Figure 2). Interestingly, digoxin and digoxigenin bind strongly to KEAP1, and they dock deeply and differentially into the central pocket of KEAP1. Hydrogen bonds formed between their C-12 hydroxy group and the Val604 residue could benefit the interaction, and the saccharide moeity of digoxin drives its binding to KEAP1, which is also supported by a hydrophobic interaction with the pocket (Figure 2). Thus, all of **1**, **10**, digoxin, and digoxigenin could bind to KEAP1, as indicated by their low docking scores (Table 2).

It has been demonstrated that NF-κB, Nrf2, and PI3K all correlate with reactive oxygen species (ROS) [29]. A crosstalk between NF-κB and Nrf2 has been evidenced by their regulation of the response to oxidative stress [22,23], and Nrf2 has been demonstrated as a downstream target of PI3K [30], indicating some potential crosstalk among NF-κB, Nrf2, and PI3K. In addition, activation of PI3K was observed from inhibition of NKA by ouabain in LLC-PK1 porcine renal epithelial cells [31], and inhibition of the PI3K pathway can yield multifaceted cancer cell-extrinsic effects to support cancer treatment [32]. Two cardiac glycosides, lanatoside C and peruvoside, were reported recently to induce human cancer cell apoptosis through the PI3K/Akt/mTOR signaling pathway, and lanatoside C was also found to bind to the key signaling proteins, including PI3K and Akt [33,34]. In addition, digoxigenin but not digoxin was found to be able to bind potentially to PI3K [14]. Following these previous investigations, both **1** and **10** were docked to the active pocket of the PI3K crystal structure (PDB ID: 6AUD), which is complexed with the inhibitor BWY. As shown in Figure 2, both **1** and **10** can fit into the binding pocket of PI3K in a similar binding pose, and their lactone unit interacts with the entrance of the PI3K pocket, while both the glycosyl group and steroid core of **1** contact with the pocket in a hydrophobic interaction (Figure 2). As a result, **1, 10**, and digoxigenin showed docking scores that are lower than that of digoxin (Table 2). However, when **1**, **10**, digoxin, and digoxigenin were docked to the pockets of the crystal structure of Akt1 complexed with a classical inhibitor (PDB entry 6CCY) or an allosteric inhibitor (PDB entry 3O96), no effective binding was observed. Compounds **1**, **10**, and digoxigenin could not reach the substrate pocket of Akt1 (6CCY), while digoxin could not form any polar interactions in the pocket. Similarly, neither pi–pi interaction (targets Trp80) nor hydrogen bond (directs Ser205) between Akt1 (3O96) and these cardenolides occurred. These results indicate that none of these cardenolides can interact with Akt1, and thus they could not target Akt1 directly to mediate cancer cell cytotoxicity. 

### 2.6. Binding between (+)-Strebloside (***1***) or Its Aglycone, Strophanthidin (***10***) and MDM2

Tumor protein P53 (p53) is a tumor suppression protein that inhibits tumor growth by inducing cancer cell apoptosis, but its function is effectively inhibited in cancer cells through its interaction with the murine double minute 2 (MDM2, a negative regulator of p53) [35]. Thus, inhibition of the p53/MDM2 interaction could lead to an enhanced antitumor potential of p53 [36], as shown by several small-molecule inhibitors, which increased the antitumor activity of p53 [37]. Previously, (+)-strebloside (**1**) was found to mediate its antitumor activity by, at least in part, inhibiting mutant p53 expression [11]. To test whether this compound directly targets the p53/MDM2 interaction, both **1** and **10**, as well as digoxin and digoxigenin, were docked to chain A of the crystal structure of MDM2 (PDB entry 4HBM). All of these compounds were found to bind moderately to the MDM2 binding pocket, as indicated by their relatively low docking scores (Table 2). Their lactone unit may form a π–π interaction with His96, which is important to the interaction of MDM2 with its inhibitors, including the co-crystal ligand, 0Y7, an inhibitor of MDM2. The steroid core and the lactone unit of both **1** and **10** adopt a similar binding mode, and hydrogen bonding could be formed between both compounds and the Tyr67 residue of MDM2. However, compared with **1** and **10**, the hydrophobicity of the steroid core of digoxin drives its interaction with MDM2, and the long saccharide moiety seems not to contribute greatly to its binding to this protein, while the steroid core of digoxigenin rotates in the binding pocket to form a hydrogen bond with the His96 residue. Thus, all of these cardenolides showed similar docking scores from their binding to MDM2 (Table 2, Figure 2).

Both (+)-strebloside and digoxin were reported to inhibit p53 by activation of the Src/mitogen-activated protein kinase (MAPK) signaling pathway, a downstream target of NKA [11,38]. Our docking results herein indicate that these cardiac glycosides and their aglycones bind to MDM2, which may compete in binding between p53 and MDM2. Thus, these cardenolides would mediate their antitumor activity through inhibition of the p53/MDM2 interaction, and they and other analogous compounds could be regarded as new inhibitors of the p53/MDM2 interaction for the design of new anticancer agents.

### 2.7. Binding between (+)-Strebloside (***1***) or Its Aglycone, Strophanthidin (***10***), and HDACs

(+)-Strebloside (**1**) was found to induce human ovarian cancer cell apoptosis [11], for which histone deacetylases (HDACs) have been proposed as important targets [39,40]. Following our previous molecular docking investigation for digoxin [14], five crystal structures from different HDAC groups were selected as the receptors for docking with (+)-strebloside (**1**) and strophanthidin (**10**), including HDAC7 (PDB entry 3C10), HDAC1 (PDB entry 4BKX), HDAC8 (PDB entry 5DC8), HDAC6 (PDB entry 5EDU), and HDAC4 (PDB entry 5ZOO). The docking profiles showed that both **1** and **10** take a similar pose in the center cavity of these proteins, and they both fit the pockets better than digoxin. However, the rigid structures of the steroid core and the lactone unit prohibit these compounds from reaching the active site, and thus the interactions of **1** or **10** with HDACs are not strong. Such low binding affinity indicates that (+)-strebloside (**1**) and strophanthidin (**10**) do not induce cancer cell apoptosis by directly targeting HDACs, but they may mediate this type of activity by other pathways, including NKA, Nrf2, PI3K, and p53 signaling.

## 3. Discussion

The cardiac glycoside digoxin has been reported for its promising antitumor activity by interacting with NKA and other targets, but it is toxic in mice, with limited promise being found from prior cancer clinical trial investigations [12,14,41]. Interestingly, (+)-strebloside was found to show more potent cancer cell cytotoxicity and lower toxicity in mice when compared with digoxin, and it targets NKA, NF-κB, and p53, to mediate its antitumor potential [9,11]. Comparison of the docking profile for (+)-strebloside (**1**) and NKA with that for digoxin shows that the detailed interactions of these compounds and NKA are different. All of the C-10 formyl and C-5 and C-14 hydroxy groups and the lactone unit of **1** contribute substantially to its interaction with NKA, and, unlike digoxigenin [14], the aglycone of **1**, strophanthidin (**10**), also binds well to this target. This different interaction may contribute to the less toxic effects observed in mice when treated with **1**. While digoxin interacts directly with NF-κB, neither **1** nor **10** seems to bind to this protein. Interestingly, both **1** and digoxin and their aglycones bind to KEAP1 (the negative regulator of Nrf2), and **1** but not digoxin, as well as the aglycone of both compounds, binds to PI3K. These results indicate that both **1** and digoxin could interact directly with Nrf2 to mediate antitumor activity, and **1** showed NF-κB inhibitory activity probably through its action on Nrf2.

Factor inhibiting HIF-1 (FIH-1) is a protein that interacts with HIF-1α to inhibit its transcriptional activity [42], and digoxin was found previously to bind strongly to FIH-1 [14]. To compare the interaction of **1** and FIH-1 with that of digoxin, both **1** and **10** were docked to FIH-1 (PDB entry 3KCX). The binding poses of **1** and **10** were found to be different. The saccharide moiety of **1** could form hydrogen bonds with the polar residues at the entry of the pocket, which enables **1** to reach deeply into the pocket. Even though the binding of **1** is different from those of digoxin and clioquinol, an inhibitor of FIH-1, the strong hydrophobic interaction between **1** and hydrophobic residues in the pocket, such as Phe100, Tyr145, and two leucines, allows **1** to penetrate the cavity deeply (Figure 2). However, **10** does not dock into the pocket, owing to the absence of the saccharide unit, and thus **10** would interact weakly with FIH-1 (Figure 2). Therefore, both (+)-strebloside and digoxin but not their aglycones could bind to FIH-1, indicating that these two cardiac glycosides may mediate their antitumor potential by targeting HIF-1.

Previously, both (+)-strebloside and digoxin were reported to inhibit p53 by targeting the Src/mitogen-activated protein kinase (MAPK) signaling pathway [11,38,43]. Herein, these two cardiac glycosides, along with their aglycones, were found to bind to MDM2, indicating that these cardenolides could directly target the p53/MDM2 interaction to exhibit antitumor activity.

NF-κB plays a key role in inflammation and human pathobiology, and both NF-κB and Nrf2 act as master regulators of the response to oxidative stress and inflammation [20,22,23]. As a result, both of these proteins have been identified as promising targets for infectious diseases [44,45,46]. In addition, the cardiac glycoside ouabain showed anti-transmissible gastroenteritis coronavirus activity via augmenting NKA-dependent PI3K_PDK1 axis signaling [47]. Therefore, by targeting NKA, NF-κB, Nrf2, and PI3K, potential infection-targeted anticancer agents may be discovered from (+)-strebloside or its aglycone.

Inhibition of the p53/MDM2 interaction has been used as a potential anticancer target, and different types of small-molecule MDM2 inhibitors have been identified as antitumor lead compounds [37,48,49]. Of these, several derivatives have been evaluated in cancer clinical trials, including nutlins, piperidinone- and pyrrolidine-containing compounds, and spirooxindoles [50]. Herein, (+)-strebloside and digoxin and their aglycones were all found to bind to MDM2, indicating that these cardenolides could be a new type of MDM2 inhibitor to show some therapeutic potential for the treatment of cancer.

Major challenges for the development of cardiac glycoside-like anticancer agents are their narrow therapeutic index, the limited selective cytotoxicity, and poor outcomes from cancer clinical trial studies. The higher toxicity against cultured human cells than the rodent cell lines and the low selectivity toward the human cancer versus non-malignant cells observed for these compounds [51,52] have hindered their developmental progress. As the most important target, NKA plays a key role in contributing to these challenges [13], as indicated by some correlations evidenced between binding to and inhibiting NKA of cardiac glycosides and between their cancer cell cytotoxicity and NKA inhibitory effects, for which the structures are critically important [10,11,41,53]. Interestingly, the cytotoxic (+)-strebloside (**1**) and digoxin were found to bind to and inhibit NKA, but their non-cytotoxic derivatives, (+)-17β-hydroxystrebloside (**8**) and (+)-17-*epi*-20,22-dihydro-21α-hydroxydigoxin did not [10,41], indicating that cardiac glycosides could be modified synthetically for their cancer cell cytotoxic and NKA inhibitory activities. Thus, searching for new structures and molecular targets for cardiac glycosides could be a promising strategy for the development of these compounds as new anticancer agents. This has been supported by a recent phase II study for PBI-05204, a *Nerium oleander* extract containing a cardiac glycoside, oleandrin, which showed that this herbal agent exhibited evidence of efficacy in metastatic pancreatic cancer patients, even though it did not improve overall survival [54]. This clinical trial may stimulate the further discovery of anticancer drugs from cardiac glycosides, and (+)-strebloside seems to be a promising lead compound.

## 4. Materials and Methods

### 4.1. Compounds and Biological Evaluation

Compounds **1**–**9** and **11** were prepared in the previous work by our group, with the cancer cell cytotoxicity and certain molecular targets also being reported [5,9,10,11]. Compound **10** was selected from the literature, which also reported its bioactivities [17].

### 4.2. Sequence Alignment and Molecular Modeling for hNKA

Following previous procedures [14], the sequence of the human NKA (hNKA) was obtained from UniProt database [55], and the crystal structures of *sus scrofa* NKA (sNKA) were selected from the Protein Data Bank (PDB) [56]. Using crystal structures 3N23, 4HYT, 4RES, and 4RET as references, the target hNKA model was produced.

### 4.3. Docking Simulation for NKA

The modeled structure of hNKA was used as the receptor, and the conformations of **1**–**11** generated by LigPrep (Schrödinger Release 2018-2: LigPrep, Schrödinger, LLC., New York, NY, USA) were used in molecular docking against the receptor by AutoDock Vina, following our previous procedure [14]. In brief, the 3D structures of **1**–**11** were built in Maestro (Schrödinger Release 2018-2: Maestro, Schrödinger, LLC., New York, NY, USA) and prepared by LigPrep from Schrodinger Suite 2018-2 (Schrödinger Release 2018-2: LigPrep, Schrödinger, LLC., New York, NY, USA). The geometric optimization was performed using the OPLS3 force field with all possible ionization states at pH 7.4 ± 0.1 created by Epik.

### 4.4. Molecular Docking for KEAP1, NF-κB, NKA, and PI3K and Akt1

AutoDock Vina [57] was used to generate the docking profiles, which were analyzed by PyMol (The PyMol Molecular Graphics System, Version 1.2r3pre, Schrödinger, LLC., New York, NY, USA ) [14]. The crystal structures of KEAP1 (PDB entry 6V6Z); the subunits of NF-κB complex, including p50 (PDB entry 1NFK), p52 (PDB entry 1A3Q), and p65 (PDB entry 2RAM); hNKA and NKA (PDB:4RET); PI3K (PDB entry 6AUD); and Akt1 (PDB entry 6CCY and PDB entry 3O96) were obtained as the receptors for the docking with **1** and **10**.

### 4.5. Molecular Docking for FIH-1, HDAC, and MDM2 

AutoDock Vina [57] was used to generate the docking profiles, which were analyzed by PyMol [14]. The crystal structures of FIH-1 (PDB entry 3KCX); HDACs, including HDAC7 (PDB entry 3C10), HDAC1 (PDB entry 4BKX), HDAC8 (PDB entry 5DC8), HDAC6 (PDB entry 5EDU), and HDAC4 (PDB entry 5ZOO); and MDM2 (PDB entry 4HBM) were obtained as the receptors for the docking with **1** and **10**.

## 5. Conclusions

In the present investigation, docking profiles for (+)-strebloside (**1**) and several derivatives and NKA have been investigated, and the docking scores were found to correlate well with their cancer cell cytotoxicity. Additionally, the docking profiles for **1** or its aglycone, strophanthidin (**10**), and several other molecular targets, including FIH-1, HDAC, KEAP1, MDM2, NF-κB, and PI3K, and Akt1 have been inspected and compared with those for digoxin and its aglycone, digoxigenin. These profiles show that both **1**, digoxin, and their aglycones could bind to KEAP1 and MDM2, and **1** and **10** also dock to the active pocket of PI3K, while **1** and digoxin interact with FIH-1. Both NF-κB and Nrf2 have been proposed as promising targets for infection [44,45,46], while HIF-1α and PI3K have been defined as the key proteins of the tumor microenvironment, targeting cancer immunotherapy and relapse problems [58]. Thus, these cardenolides could be developed as p53/MDM2 interaction- and tumor microenvironment-targeted cancer chemotherapeutic agents, and (+)-strebloside seems to be more promising than digoxin for this purpose. This is due particularly to its more potent cancer cell cytotoxicity, lower mouse toxicity, and potential binding to PI3K when compared with digoxin.

## Figures and Tables

**Table 1:** Docking scores from the binding to NKA and cytotoxicity of strebloside (**1**) and its derivatives (**2**–**11**).^a.^

Compd.	Docking Score (kcal/mol)		Cytotoxicity	Compd.	Docking Score (kcal/mol)		Cytotoxicity
	Average	Minimal			Average	Minimal	
**1**	−10.4	−11.8	0.17 ^b^ 0.01 ^c^	**7**	−7.8	−9.7	>20 ^b^
**2**	−9.7	−11.5	0.47 ^b^	**8**	−8.7	−11.0	>20 ^b^
**3**	−10.0	−11.6	0.16 ^b^	**9**	−8.8	−9.8	>20 ^b^
**4**	−9.3	−10.3	0.69 ^b^	**10**	−9.6	−11.4	0.27 ^c^
**5**	−10.3	−11.4	0.09 ^b^	**11**	−9.6	−10.7	>20 ^b^
**6**	−8.8	−11.0	1.2 ^b^				

^a^ Docking scores (kcal/mol) from binding between **1**–**11** and NKA (PDB entry 4RET) calculated by AutoDock Vina. IC_50_ (µM) values for cytotoxicity against human ^b^ HT-29 colon and ^c^ A549 lung cancer cells [5,9,17].

**Table 2:** Docking scores (minimal, kcal/mol) of **1**, **10**, digoxin, and digoxigenin calculated from their binding to FIH-1, KEAP1, MDM2, NF-κB, NKA, and PI3K.

Compd.	FIH-1	KEAP1	MDM2	NF-κB p50	NF-κB p52	NF-κB p65	NKA	PI3K
**1**	−9.3	−8.9	−8.3	−7.4	−7.0	−6.9	−11.8	−7.0
**10**	−9.9	−9.7	−8.1	−7.2	−7.1	−7.1	−11.4	−6.9
digoxin	−10.6	−10.0	−8.0	−9.2	−8.5	−8.8	−12.5	−6.4
digoxigenin	−9.2	−9.1	−9.0	−7.7	−6.7	−7.1	−10.0	−7.2

## Data Availability

The data presented in this study are available on request from the corresponding author, but they are not publicly available due to the requirements of ongoing research.

## Sample Availability

A sample of compound **10** is not available from the authors.
